# Astragalus *sinicus* Incorporated as Green Manure for Weed Control in Corn

**DOI:** 10.3389/fpls.2022.829421

**Published:** 2022-04-29

**Authors:** Silin Liu, Zhiyi Ma, Ying Zhang, Zhongwen Chen, Xiao Du, Yinghui Mu

**Affiliations:** ^1^College of Agriculture, South China Agricultural University, Guangzhou, China; ^2^School of Electrical and Mechanical Engineering, Zhongkai University of Agriculture and Engineering, Guangzhou, China; ^3^Scientific Observing and Experimental Station of Crop Cultivation in South China, College of Agronomy/Ministry of Agriculture and Rural Affairs, Guangzhou, China

**Keywords:** milk vetch, goosegrass suppression, allelopathy, corn growth, phytotoxicity

## Abstract

*Astragalus sinicus* L. (milk vetch), one of the most widespread green manure species, is widely planted in the temperate zone. *Eleusine indica* L. (goosegrass), a serious annual weed in the world, has evolved resistance to some non-selective herbicides. The use of milk vetch as green manure for weed control in paddy fields was proposed. Aqueous extracts of milk vetch are known to exert a different level of phytotoxicity on weeds and crops. Phytotoxic substances contained in green manure were released into the soil by leaching at the initial stage and decomposition at the later stage after the return of green manure. Considering the need for searching new sustainable strategies for weed control, a question arises: “if milk vetch could be applied in goosegrass control, which stage is the most important to control goosegrass after milk vetch returned to the field, and at the same time, will the subsequent crop, corn (*Zea mays* L.), be affected by the side effects from milk vetch phytotoxicity?” In this study, the potential of milk vetch for goosegrass control was approached by repeated laboratory experiments, which include the aqueous extract experiment, decomposed experiment, and pot experiment. The effects of milk vetch returning to the field on maize were simulated by a pot experiment. The extract of milk vetch could significantly inhibit the germination of goosegrass at 2% concentration, and the inhibition enhanced with the increase of concentration. In the decomposed liquid experiment, decay time within 15 days, with the increase of decay days or concentration, goosegrass inhibition effect of decomposed liquid was enhanced. When decay time was more than 15 days, the inhibition ability of the decomposed liquid to goosegrass decreased. According to the RI accumulated value, aqueous extract and decomposed liquid have a “hormesis effect” on the germination and growth of goosegrass. Pot experiment proved that the addition of 1–10% (w/w) of milk vetch significantly reduced the germination and growth of goosegrass. On the contrary, the comprehensive analysis showed that the participation of milk vetch was conducive to the growth of corn. Our results constitute evidence that the incorporation of milk vetch into the soil could be a feasible practice to reduce weed infarctions in the corn-based cropping system.

## Introduction

Goosegrass is an annual gramineous weed, which is a worldwide weed harmful to crop growth in agricultural production (Zhang et al., 2021). In some suitable areas, it becomes a malignant weed due to its strong fecundity and resistance (An et al., 2014). In recent years, the widespread and excessive use of synthetic herbicides for weed control has led to many problems such as the development of herbicide-resistant weed species, damage to soil microecology, and environmental pollution. There is an urgent need to find other forms of weed control that can reduce the use of synthetic herbicides (Tesio and Ferrero, 2010). Milk vetch is a biennial herb of the leguminous family astragalus, and an important green manure crop. Previous studies showed that applying milk vetch could improve rice yield and reduce the use of chemical fertilizers (Chen et al., 2020; Zhou G. P. et al., 2020). Researchers found that the returning of milk vetch changed soil microbes and has the greatest potential to improve crop productivity as well as increase in corn yield (Tao et al., 2017). Studies have shown that planting milk vetch in winter fallow fields can improve soil fertility, reduce compound fertilizer application, improve soil physical and chemical properties, and preserve soil and water (Yang et al., 2014; Zhou et al., 2019; Zhou X. et al., 2020). In addition, intercropping of milk vetch could improve the quality, yield, and pest resistance of target crops (Bista and Dahal, 2018). Researchers found that incorporating milk vetch in paddy fields could significantly reduce the density of weed seed banks and significantly increase the species diversity and evenness of weed seed banks, which indicated that milk vetch returning to the field has the potential to control weed (Tang et al., 2016). Researchers found that the incorporation of milk vetch can significantly suppress the germination of summer weeds in paddy fields (Utami et al., 2020). Therefore, the research focus of milk vetch is on its effects on crop growth, soil physical and chemical properties, diseases, and insect pests, while there are a few studies on its inhibitory effect on weeds, especially on dryland weed control. Meanwhile, the physiological mechanism of how milk vetch inhibits weed growth is still unclear. There are many studies on the effect of returning leguminous crops on weeds. Researchers based their findings on field tests that faba bean (*Vicia faba* L.) could exert weed control when used as green manure, and the weed inhibition ability was highest at the time of faba bean incorporation into the field (Álvarez-Iglesias et al., 2018). However, He et al. (2020) found that corn seed germination and the lengths of radicle and plumule were lower than those of the control, particularly with 1% faba bean aqueous extract concentrations. Moreover, incorporating faba bean as green manure, the aboveground biomass of corn at the heading stages was negatively reduced by the whole plant, aboveground part, and stem (He et al., 2020). Milk vetch and spring corn rotation is an important tillage system. Previous studies have revealed that incorporating milk vetch into the soil can suppress weed infarctions. It is the premise of scientific and rational tillage to fully study the phytotoxicity effect of green manure on the main economic crops. Thus, it is necessary to further study whether the return of milk vetch to the field affects the corn-based tillage system. In this study, three goosegrass control laboratory experiments were designed with milk vetch as the material, based on previous studies and field observations, to find out the optimal decay time of allelochemicals in the decomposition process of milk vetch, and to clarify the impact of milk vetch returning on the growth of corn. It provides a theoretical basis for rational utilization and development of milk vetch in agricultural production.

## Materials and Methods

### Plant Material

Milk vetch (*Astragalus sinicus* L.) seeds for the experiment were provided by Royal Garden Greening Engineering Co., Ltd. In July 2020, milk vetch was seeded in an artificial climate incubator (temperature 23 ± 2 (temperature %, 12 h/12 h light–dark cycle) and kept the soil moist. In September of the same year, milk vetch was harvested, air-dried, and crushed under natural conditions, sealed, and stored at room temperature for later use. Goosegrass (*Eleusine indica* L.) seeds were collected in the Experimental Base of South China Agricultural University (23°14’18.42′′.42re c°38’8.0642re collected in the Experime“Black pearl waxy corn.”

### Experiment and Treatment Design

This study includes milk vetch aqueous extract experiment, decomposed liquid experiment, and pot experiment. In the aqueous extract experiment, we used dry milk vetch powder as the material, weighed 20 g of powder, put in a bottle, added 500 ml sterilized ultra-pure water, and soaked in a shaker for 48 h with 200 rpm. The aqueous extract was filtered by filter paper and centrifuged at 12,000 rpm for 10 min. The supernatant was filtered and the 0.22 μm microporous membrane was used to remove bacteria. The concentration of the undiluted aqueous extract was set as 100% and diluted with sterilized ultra-pure water into 2, 5, 30, and 80% as the treatment solution. We used 2.2% sodium hypochlorite to disinfect the goosegrass seeds for 10 min, and adopted the double-layer filter paper method, inoculated with 30 seeds of goosegrass into Petri dishes, treated them with the treatment solution. Sterile water treatment was used as a control (CK), each treatment was set up 5 replicates. Placed the inoculated Petri dishes in an artificial climate incubator (KES) at a temperature of 26 ± 2°C, a relative humidity of 75%, and a light–dark cycle of 12 h/12 h. Replenished water and recorded the germination number every day. The emergence of the radicle was taken as the indicator of germination. When the germination rate remained unchanged for 3 days, the recording was stopped.

In the decomposed experiment, we used milk vetch powder as material, mixed it according to the following ratio: powder, fresh paddy soil, and ultra-pure water (1:1:30 by weight) and put them into a plastic bottle with an inner lid. Soil solution without powder was used as the control (CK), and placed in a constant temperature shaker at 28°C for 200 rpm for 2, 3, 7, 15, 20, 30, and 42 days to obtain the decomposed liquid. The decomposed liquid from 15 days ago was used for the test immediately after decomposition, and the decomposed liquid from the subsequent time points (i.e., 20, 30, and 42 days) was stored in a -20ed in asequtor and was used for the test together after the last decomposition. The decomposed liquid and the control were filtered by filter paper and centrifuged at 12,000 rpm for 10 min. The supernatant was filtered by 0.22 μm microporous membrane, and the obtained decomposed liquid was diluted with sterilized ultra-pure water into 2, 5, 30, and 80% concentration gradient treating solution. The next steps were similar to the aqueous extract experiment.

In the pot experiment, according to the dry weight yield of milk vetch 11.25 t/hm and the soil bulk density of about 1.3 g/cm^3^, assuming that the tillage depths of no-tillage and rotary tillage are 1 and 6 cm, respectively, the milk vetch straw ratio is around 1.44–8.66%. We used milk vetch powder as material, mixed it well according to the mass ratio of powder:substance (Jiffy, consists of peat soil, coconut bran, vermiculite, and perlite) = 1:100, 3:100, 6:100, and 10:100, and the mixture was put in the flowerpot, and matrix soil without powder was used as the control (CK). Inoculated 30 goosegrass seeds or nine corn seeds into the flowerpot, each treatment was set up five replicates. The seeded pots were placed on the terrace, the average temperature during the experiment being 24°C, and the relative humidity being 85%. To ensure the authenticity of the results, all the management and environmental condition were maintained as consistent as possible.

### Data Collection

In the germination stage of the aqueous extract and decomposed liquid experiment, we determined six germination indicators, namely, germination potential, germination rate, germination index, vital index, plant height, and fresh weight. Ten seedlings with uniform growth were selected from each treatment to measure plant height. A total of 20 plants were randomly selected from the five replicates for three replicates to determine the fresh weight of the plants (The number of samples was reduced by a certain proportion, and the final result was multiplied by the corresponding multiple after weighing when the sample was insufficient, same as the goosegrass of pot experiment). In the pot experiment, the seed germination rate of corn and goosegrass was recorded 8 days after inoculation, because no new seeds germinated after 8 days. Fifteen days after inoculation, 10 seedlings of goosegrass and 5 seedlings of corn with uniform growth were selected from each treatment to measure plant height. Five corn plants with uniform growth were selected from each treatment to measure fresh weight. At the same time, biochemical indicators, photosynthetic characteristics, and morphologic photographs of goosegrass and corn were recorded. Germination-related indicators were calculated according to the following formula:


G⁢p-n⁢u⁢m⁢b⁢e⁢r⁢o⁢f⁢g⁢e⁢r⁢m⁢i⁢n⁢a⁢t⁢e⁢d⁢s⁢e⁢e⁢d⁢s⁢a⁢t⁢ 2⁢dn⁢u⁢m⁢b⁢e⁢r⁢o⁢f⁢t⁢e⁢s⁢t⁢e⁢d⁢s⁢e⁢e⁢d⁢s×100%;

$$G\,r-\frac{n\,u\,m\,b\,e\,r\,o\,f\,n\,o\,r\,m\,a\,l\,g\,e\,r\,m\,i\,n\,a\,t\,e\,d\,s\,e\,e\,d\,s}{n\,u\,m\,b\,e\,r\,o\,f\,t\,e\,s\,t\,e\,d\,s\,e\,e\,d\,s}\times 100\%;$$

V⁢i-s×G⁢i,(s⁢m⁢e⁢a⁢n⁢s⁢p⁢l⁢a⁢n⁢t⁢h⁢e⁢i⁢g⁢h⁢t⁢h⁢e⁢r⁢e);

$$Gi-\sum \left(\frac{G\,t}{D\,t}\right),(Gt:Germinationnumberatdifferentday,Dt:Thestatisticalnumberofdays);$$

Gr: Germination rate; Gp: Germination potential; Gi: Germination index; Vi: Vital index.

The allelopathic effects of milk vetch were evaluated using means of response index (RI). When T ≥ C, RI = 1-C/T; T < C, RI = T/C-1 (C: result of CK; T: result of treatment). RI > 0, indicating allelopathic promotion; RI < 0, indicating allelopathic inhibition; absolute value was consistent with the degree of allelopathy (Williamson and Richardson, 1988). RI accumulate values of each indicator were calculated and the allelopathic effects of milk vetch were evaluated comprehensively. RI accumulate values reached -6, indicating that germination and growth of weeds were totally inhibited. In the second-leaf stage of corn in the pot experiment, the last fully expanded leaf of goosegrass and corn seedlings was taken to determine the protective enzyme activity, content of soluble protein, and malondialdehyde (MDA) content. Superoxide dismutase (SOD) activity was measured by the nitroblue tetrazolium (NBT) reduction method, peroxidase (POD) activity was measured by the colorimetric method, and catalase (CAT) activity was measured by the guaiacol method (Zhang et al., 2017). Soluble protein content was measured by Coomassie’s brilliant blue staining (Goharrizi et al., 2021). MDA content was measured by the TBA method and the root activity was measured by the 2,3,5-Triphenyte-trazoliumchloride (TTC) method (Xiao et al., 2021). Photosynthesis and fluorescence-related indexes, namely, net photosynthetic rate (Pn), transpiration rate (E), stomatal conductance (gtc), intercellular carbon dioxide concentration (Ci), instant water use efficiency (iWUE), and maximal quantum yield of PSII (Fv/Fm), PSII actual photochemical quantum efficiency (φPSII), electron transport rate (ETR), photochemical quenching (qP), and non-photochemical quenching (NPQ), were measured by an LI-6,800 photosynthesis measurement system (*LI*-COR, United States). The last fully expanded leaf of 3 uniform corn seedlings at the fourth-leaf stage was selected from 5 replicates of each treatment and the parameters related to photosynthesis and fluorescence were measured. The chlorophyll content was expressed by the Soil and Plant Analyzer Development (SPAD) value, which was collected by SPAD-502 plus (Konica Minolta, Inc.). The last fully expanded leaf of a fourth-leaf corn seedling was selected from each of the 5 replicates of each treatment, and the SPAD values of the upper, middle, and lower parts of the leaf were measured, and their average value was calculated as the final SPAD value. Root and leaf area analysis was performed using GXY-A, a scanner produced by Zhejiang Tuopu Yunnong Technology Co., Ltd. One corn seedling was selected from each of the five replicates of each treatment, and all roots and leaves were cut and scanned by GXY-A. Root morphologic parameters were analyzed by WinRHIZO, and leaf area was calculated by Photoshop. Both are well known for image processing.

### Statistical Analysis

The data of experiments were analyzed by one-way ANOVA. We verified normality and homogeneity of variance using the Shapiro–Wilk test and Levene’s test, respectively. Significant differences were further compared using the *post hoc* Fisher LSD test. SPSS 24 was used for significance analysis of the data, which were expressed as mean ± standard deviation. GraphPad Prism 7 was used to plot.

## Results and Discussion

### Effect of Milk Vetch Aqueous Extract on Goosegrass

**FIGURE 1:**
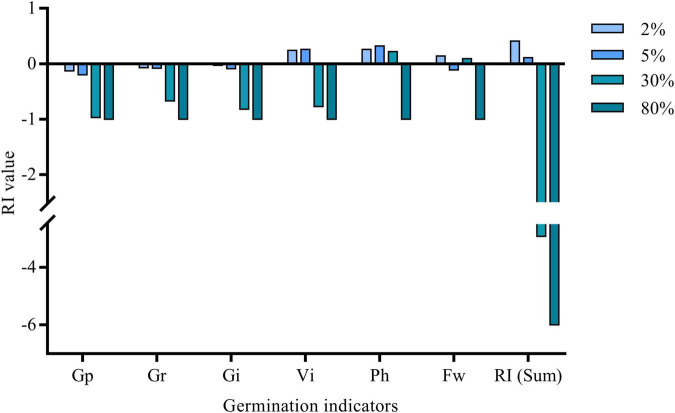
RI value of milk vetch aqueous extract on germination and growth of goosegrass. Gr, Germination rate; Gp, Germination potential; Gi, Germination index; Vi, Vital index; Ph, Plant height; Fw, Fresh weight; RI (Sum), The sum of the respond index value of Gp, Gr, Gi, Vi, Ph, and Fw.

With the increase in the concentration of the milk vetch–leached liquor (Figure 1), the inhibitory effect of the aqueous extract on Gp, Gr, and Gi was enhanced. Low concentration leached liquor (≤30%) can improve the plant height of goosegrass, while the vital index was improved with less than 5% aqueous extract treatment. The fresh weight of goosegrass did not show an obvious rule. Researchers studied the allelopathic effects of eucalyptus leachates on three tree species and found that foliage + litter and foliage leachates even increased fresh weight and dry weight of *Leucaena leucocephala* and *Schefflera octophylla*, respectively (Song et al., 2019). When the concentration of milk vetch aqueous extract reached 80% (Supplementary Table 1), all the goosegrass seeds in the test could not germinate so the RI value reached -6. The variety of goosegrass germination indicators were dose dependent. Many studies reveal that the allelopathic effect of a plant extract or a leachate was dependent of its concentration (Chen B. M. et al., 2018; Eladel et al., 2019; Ojija et al., 2019). According to the RI accumulate value, milk vetch leached liquor has a “hormesis effect” on germination and growth of goosegrass. Only from the point of view of germination, the results of the aqueous extract experiment were consistent with the previous studies on the effects of *Hyptis suaveolens* (L.) Poit on the *Parthenium hysterophorus* I. and *Senna uniflora* (Mill.) H.S. Irwin (Kumari and Prasad, 2018). Allelopathic substance affects different plant receptors in different directions, some for germination and some for growth. Returning eucalyptus leaves as green manure can inhibit the germination of representative weeds in the field (Puig et al., 2013). In the aqueous extract experiment, depending on the RI value of germination and growth indicators of goosegrass, we can conclude that milk vetch aqueous extract mainly affected the germination process of goosegrass. We did not find significant negative effects of aqueous extract on plant height and the fresh weight of goosegrass seedlings. Aqueous extract within 30% even increased the plant height of goosegrass, and 2 and 30% concentration can also increase the fresh weight.

### Effect of Different Decay Time Decomposed Liquid of Milk Vetch on Goosegrass

**FIGURE 2:**
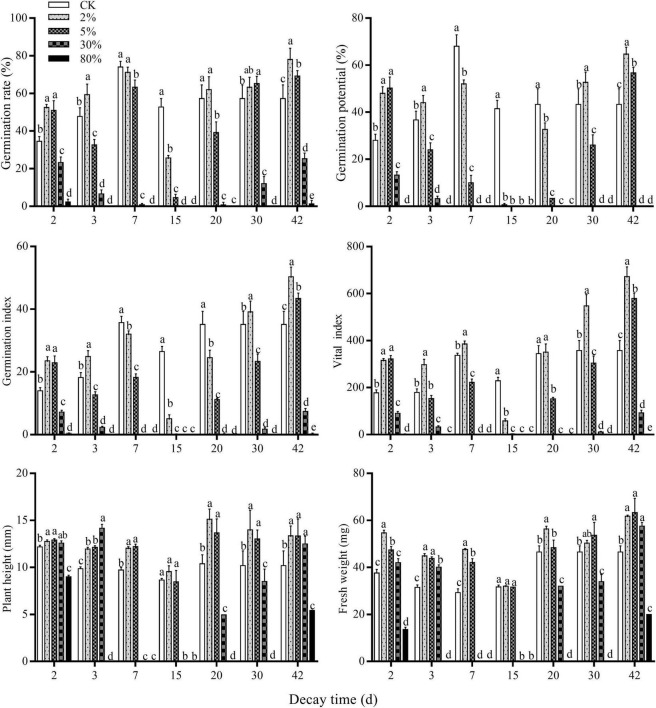
Effect of different decay time milk vetch decomposed liquids on germination and growth of goosegrass. Different letters above each group of the bar chart denote significant difference among different treatments of decomposed liquid at *P* < 0.05 by LSD test, respectively. Bars represent 10, 3, and 5 repetitions ± SD in plant height, fresh weight, and germination-related parameters, respectively.

**FIGURE 3:**
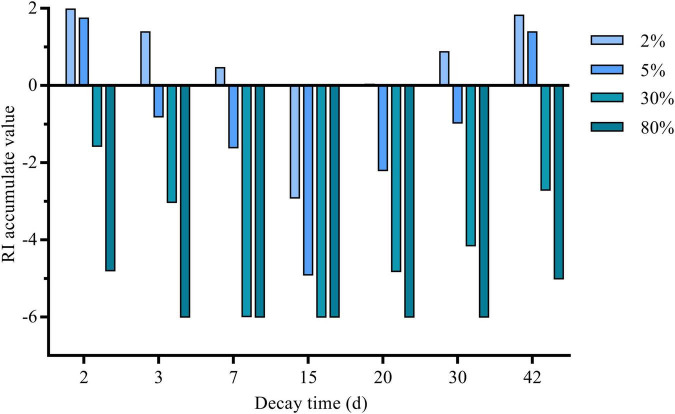
RI accumulated value of milk vetch decomposed liquids on goosegrass. RI: Respond index.

The release of allelochemicals through decomposition is an important way for green fertilizer to exert the effect of weed control. The germination-related indicators (Figure 2), namely, germination potential, germination rate, and germination index were significantly improved in 2 days decomposed liquid of ≤ 5%, and 3 days decomposed liquid of 2%. All the germination-related indicators were inhibited in 7 and 15 days decomposed liquids of all the concentration treatments. The 15 days decomposed liquid had the highest goosegrass-inhibited potential among all the decay times. When the decay time was greater than 15 days and less than 30 days, germination-related indicators went up gradually at low concentrations treatment (≤5%). When it was 42 days, decomposed liquids less than 30% could significantly improve all the indicators of goosegrass. None of the seeds germinated at 80% decomposed liquid except those with 2 and 42 days treatment. The vital index of goosegrass seeds was affected by the interaction of germination index and plant height. The low concentration decomposed liquids of 2 and 45 days treatment can significantly improve the vital index of goosegrass, while 2% decomposed liquids of 3, 7, 20, and 30 days can improve the vital index. A 15 days decomposed liquid can significantly inhibit the vital index of goosegrass among all the concentration treatments. The change of plant height and fresh weight of the goosegrass seedlings have a similar tendency. On 2, 3, and 42 days, both plant height and fresh weight increased when the concentration of decomposed liquids was less than 30%, but with the increase in concentration, plant height and fresh weight were significantly inhibited. The plant height and fresh weight were increased by 7, 20, and 30 days decomposition solution at low concentration (≤5%), but plant height and fresh weight were significantly inhibited with the increase of concentration. Plant height and fresh weight have an insignificant change in 15 days decomposed liquid of low concentration treatment but were significantly inhibited at high concentration treatment (≥30%). In the field of allelopathy research, we often see that the law of allelopathy among plants is summarized as low promotion and high inhibition, which simply means the “hormesis effect.” *Carex thunbergii* extracts increased the germination rate of *Lolium perenne* at low concentrations and showed an allelopathic inhibition effect at a high concentration (Zhang et al., 2019). In conclusion, the phytotoxicity potential of milk vetch decomposed liquid on goosegrass varied with the decay time, which reached the peak at 15 days and was dose dependent. In the allelopathic effect experiment of soybean straw decomposition on mung bean, researchers found that in four periods (0, 7, 14, and 21 days), 14 days of decomposition could produce a variety of chemical substances and significantly inhibit mung bean, whereas decomposition for 21 days reduced the inhibitory effect (Azhar et al., 2018). The decomposition patterns and the persistence of allelopathic chemicals are different in different plants. Researchers found a rule in the cereal rye cover crop decomposition studies that the phenolic acids released from cereal rye increased in soils during the first 3–7 days after cereal rye termination and then decreased to initial concentrations after 56 days (Otte et al., 2020). The allelopathic ability increased with the increase of decomposition time before 15 days of decomposition, and the allelopathic inhibition ability decreased after 15 days of decomposition, which was similar to a previous research. As shown in Figure 3, the intensity of the allelopathy of the decomposed liquid was determined by the concentration and the decay time.

### Effect of Simulation of Milk Vetch Simulated Returning to the Field on the Germination and Growth of Goosegrass

#### Different Ratios of Milk Vetch Straw and Soil Treatments on Goosegrass Morphologic Properties

**FIGURE 4:**
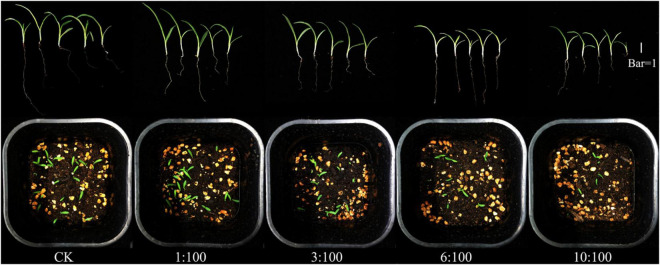
Effect of different milk vetch straw and soil ratios on goosegrass.

**FIGURE 5:**
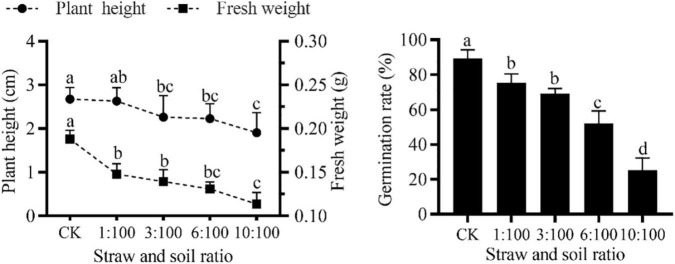
Effect of different ratios of milk vetch straw and soil on germination and growth of goosegrass. All data were subjected to analysis of variance with *post hoc* LSD test. Different letters denote significant difference among different treatments at *P* < 0.05. Symbols represent 10, 3, and 5 repetitions ± SD in plant height, fresh weight, and germination rate, respectively.

Only from the weed control perspective (Figure 4), the significant weed inhibition effect can be seen intuitively with the input of milk vetch straw in different straw and soil ratios. The germination rate, plant height, and fresh weight of goosegrass (Figure 5) were significantly inhibited by the incorporation of milk vetch straw. A 1:100 straw-to-soil ratio has an insignificant effect on plant height of goosegrass, with the ratio going up, plant height was significantly inhibited. Fresh weight of goosegrass was inhibited significantly among all the ratios treatment of the experiment. The germination and growth-related indicators of goosegrass did not improve in the pot experiments even in the lowest straw proportion treatment. In a study of weed control on the corn field, researchers found that the incorporation of faba bean as green manure can reduce the germination, root and bud elongation, and aboveground biomass of major weeds in the corn fields, and a higher proportion of faba bean treatment showed stronger weed inhibition potential (Álvarez-Iglesias et al., 2018). Submerged macrophytes (*Ceratophyllum demersum* L.) could allelopathically inhibit the growth of *Chlorella vulgaris* Beij., the effects of which were concentration-dependent (Dong et al., 2019). The return of milk vetch to the field reduced the occurrence of goosegrass and inhibited the growth of the germinated weeds, which is worth further study. The researchers used the *eucalyptus globulus* leaves as green manure and isolated two allelopathic substances after returning to the field, which inhibited the germination and growth of weed (Puig et al., 2018).

#### Different Ratios of Milk Vetch Straw and Soil Treatment on Photosynthesis-Related Properties of Goosegrass

Compared to the CK, different straw and soil ratio treatments can significantly inhibit the net photosynthesis rate (Pn) and instant water use efficiency (iWUE) of goosegrass. Allelochemicals cannot only inhibit the growth establishment but also significantly decrease the photosynthetic rate of lettuce (Nasrollahi et al., 2018). Treatments of 3:100 and 6:100 ratios significantly reduced the transpiration rate **(E)** of goosegrass, while 1:100 and 10:100 treatments did not significantly reduce the transpiration rate, but slightly decreased. Different straw and soil ratio treatments can decline the stomatal conductance (gtc) of goosegrass, and in the high straw and soil ratio treatments, the change reached a significant level. Different straw and soil ratio treatments can improve the intercellular carbon dioxide concentration (Ci) of goosegrass, and in 1:100 and 10:100 treatments, the change of intercellular carbon dioxide concentration reached a significant level. Chen S. et al. (2018) reported that the decomposing leaf litter of *Leucaena leucocephala* can decrease the photosynthetic rate, transpiration rate, and stomatal conductance, and continuously increase the intercellular carbon dioxide concentration, which was consistent with this study. Researchers speculated that in plants under stress, the photosynthesis characteristic was inhibited, which leads to a lower net photosynthetic rate and higher intercellular carbon dioxide concentrations (Bao et al., 2020).

Chlorophyll fluorescence serves as a sensitive indicator of the functional state of photosynthetic apparatus in chloroplasts (Kalmatskaya et al., 2019). Different milk vetch straw and soil ratios have no significant impact on the maximal quantum yield of PSII (Fv/Fm). The highest value of PSII actual photochemical quantum efficiency (φPSII) and electron transport rate (ETR) were recorded in the 6:100 treatment. Compared to the CK, the φPSII and ETR of goosegrass were significantly suppressed, except for the 6:100 treatment. The lowest photochemical quenching (qP) value was recorded in the 1:100 treatment, while the highest value was recorded in the 6:100 treatment, and the rest treatments had no significant effect compared with CK. Wang et al. (2018) on the study of *Dracontomelon duperreanum* leaf litter impacted the photosynthesis of *Microcystis aeruginosa* found that the growth rate of *Microcystis aeruginosa* in response to different extract concentrations was consistent with changes in the photosynthesis efficiency (alpha), maximal relative electron transport rate, and maximal quantum yield of PSII. Compared with our study, although it was slightly different from previous studies in some indicators, it could be found that the photosynthesis of goosegrass was adversely affected in general.

When plants live under stress, it is often accompanied by an increase in non-photochemical dissipation of excitation energy, NPQ (Doğru and Çakirlar, 2020). The lowest non-photochemical quenching (NPQ) value was recorded in CK, while the highest value was recorded in 6:100 treatment, and the rest treatments were significantly improved compared with CK. The non-photochemical quenching significantly improved in all treatments, which indicated that the plant’s photosynthetic system has been adversely affected (Supplementary Table 2). According to previous studies, researchers hold the view that high NPQ values indicate a disadvantaged condition where plants are living in Kalmatskaya et al. (2019) and Gao et al. (2020).

#### Different Ratios of Milk Vetch Straw and Soil Treatment on the Biochemical Properties of Goosegrass

**FIGURE 6:**
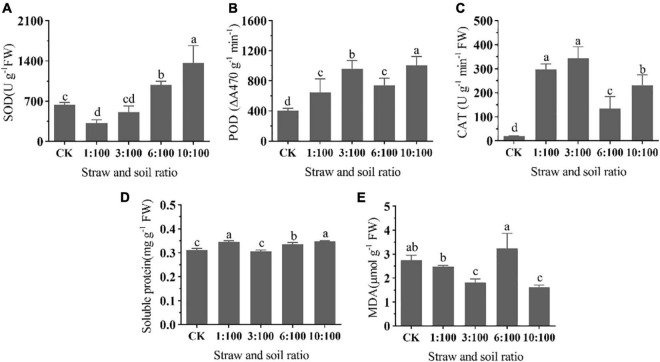
Effect of different ratios of milk vetch straw and soil on SOD activity **(A)**, POD activity **(B)**, CAT activity **(C)**, soluble protein **(D)**, and MDA **(E)** content of goosegrass. All data were subjected to analysis of variance with *post hoc* LSD test. Different letters denote significant difference among different treatments at *P* < 0.05. Bar chart represents mean values of 5 replicates ± SD.

The SOD activity of goosegrass seedlings was significantly declined at 1:100 treatment and gradually improved with the increase of straw ratio. SOD activity increased significantly when the percentage of straw was more than 6% (Figure 6A). The change tendency of POD and CAT activity were synchronous, which were significantly improved at all straw and soil ratio treatments. Compared to other treatments, POD activity at 6:100 treatment was slightly declined. CAT activity at a high ratio treatment (≥6:100) was relatively lower than the rest of the treatment (Figures 6B,C). Researchers of the studies on allelopathic effect of *Bidens pilosa* leachates on *Pteris multifida* found that superoxide dismutase (SOD) and catalase (CAT), as well as guaiacol peroxidase enhanced with the increase in leachate concentration (Zhang et al., 2016). Phytotoxic substances (cinnamic acid) can induce the production and accumulation of ROS, decrease root and shoot length, fresh and dry weights, and photosynthetic pigments (Yadav et al., 2019). In this study, we did not measure the ROS content, but from the change in antioxidant enzyme activity, we suspected that decomposing liquid may increase the ROS content in plants. All of the treatments can significantly improve the soluble protein except for the 3:100 treatment (Figure 6D). A 6:100 straw and soil ratio treatment can increase the MDA content of goosegrass, but the level did not reach a significant level. The rest treatment can significantly decrease the MDA content of goosegrass except for the 1:100 treatment (Figure 6E). The photosynthesis-related indexes and chlorophyll content of the 6:100 treatment were relatively poor, which may be related to MDA content. The change in the protective enzyme system means that peroxides and superoxides were produced during the decomposition of milk vetch. According to the change of POD, CAT, and MDA, we speculated that the content of MDA was mainly affected by peroxide. As for why the change of MDA content was not parallel with the milk vetch proportion, a previous study found that the release of allelochemicals by the interaction of green manure and microorganism is an important process of green manure returning to the field. Ping et al. (2015) found that different proportions of corn straw returned to the soil can affect the community composition of soil microbes, but the signatures (PLFAs) were non-linear with straw and soil ratios, which may be associated with the non-linear change in peroxide-related enzyme activity as well as MDA content in our study. Coumarins are widely distributed substances in plant species that promote phytotoxic effects, allowing them to be exploited as herbicides less harmful to the environment. The researchers found that coumarin can inhibit crop germination, reducing its biomass, and root and shoot growth. Subsequent studies have shown that coumarin is cytogenotoxic due to its damage to the cell cycle and the occurrence of chromosomal abnormalities. However, it does not cause lipid peroxidation (Govêa et al., 2020), most of Govêa et al.’s findings are consistent with this study.

### Effect of Simulation of Milk Vetch Simulated Returning to the Field on the Germination and Growth of Corn

#### Different Ratios of Milk Vetch Straw and Soil Treatments on the Morphologic Properties of Corn

**FIGURE 7:**
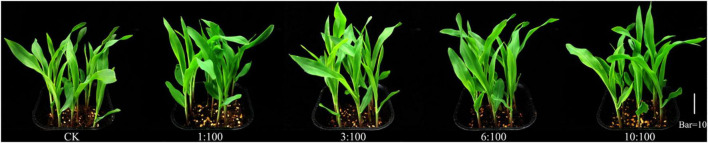
Effect of different ratios of milk vetch straw and soil on the growth of corn.

**FIGURE 8:**
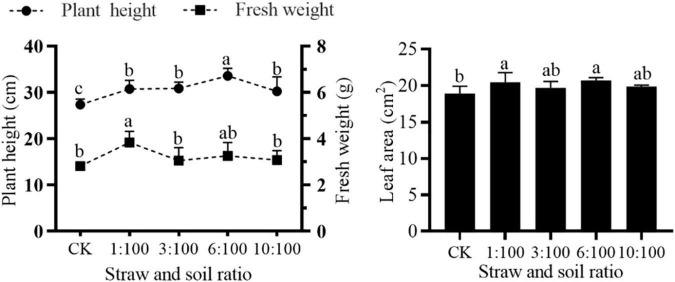
Effect of different ratios of milk vetch straw and soil on plant height, fresh weight, and leaf area of corn. All data were subjected to analysis of variance with *post hoc* LSD test. Different letters denote significant difference among different treatments at *P* < 0.05. Symbols represent 5 repetitions ± SD.

Many studies have reported that the incorporation of milk vetch can improve the yield of the main crop and the productivity and sustainability of soil (Yang et al., 2013). Before returning green manure to the field for weed control, it is necessary to assess the duration of the early effects of green manure incorporation into the soil and its short-term effects on germination and growth of the main crop and some accompanying weeds (Puig et al., 2013; Álvarez-Iglesias et al., 2018). The effect of green manure allelopathy in corn must be taken into account for putative field management of green manure for weed control. Consequently, a pot experiment on the phytotoxic effects of green manure was designed to establish a relay planting of corn after the incorporation of milk vetch into the soil. Root morphogenesis at the seedling stage is very important to growth and yield at the later stage. Compared to the CK, all the treatments can improve the root system of corn except for the total length of corn at 10:100 condition and branch points of corn at 3:100 condition. Incorporating a certain percentage of milk vetch into the soil can significantly improve all the root systems of corn except total length and branch points (Supplementary Table 3). Root growth and development were closely related to reactive oxygen species (ROS), the reduced ROS content resulted in defective root growth of cucumber (Huang et al., 2020). Whether corn root growth is exactly affected by ROS and the influence of certain milk vetch ratios for root growth needs further study. Visually, adding vetch straw can promote the growth of corn seedlings (Figure 7). All the treatments can significantly improve the plant height of corn (Figure 8). Application of green manure together with out-of-season corn crop promoted the highest plant height and soybean yield-related traits in succession (Genovesi et al., 2019). The fresh weight of corn could be increased by different milk vetch proportion treatments, and the change level reached a significant level at 1:100 ratio treatment. All the treatments can improve the leaf area of corn, and the change reaches a significant level at 1:100 and 6:100 treatments. The return of green manure provides a breeding ground for subsequent crops, which will be a benefit to the growth and development of crops. Previous studies can also prove this point. Crop straw combined with green manure crops improved the germination rate, growth process of corn plants, and contributed to the better establishment of generative organs, elements of the structure of the yield (Sendetsky, 2018). In conclusion, milk vetch returning was a possible practice, which has the potential to improve the growth of corn seedlings.

#### Different Ratios of Milk Vetch and Soil Treatment on Chlorophyll Content and Photosynthesis-Related Properties of Corn

The incorporation of milk vetch was beneficial to all the photosynthesis properties of corn except for transpiration rate at 1:100 treatment and instant water use efficiency at 10:100 treatment. The net photosynthetic rate (Pn), transpiration rate (E), and stomatal conductance (gtc) of maize were significantly improved when the ratio of milk vetch was more than 1:100. Different milk vetch and soil ratios can improve the intercellular carbon dioxide concentration (Ci) of corn, but the change did not reach a significant level. Instant water use efficiency (iWUE) can be improved when the milk vetch proportion is less than 6%, and the change in 1:100 treatment reached a significant level. Not only green manure, crop stalks can also play a similar role. Researchers found that an appropriate ratio of corn straw in the substrate could significantly improve the photosynthetic parameters and chlorophyll content of tomato seedlings (Chen S. et al., 2018).

**FIGURE 9:**
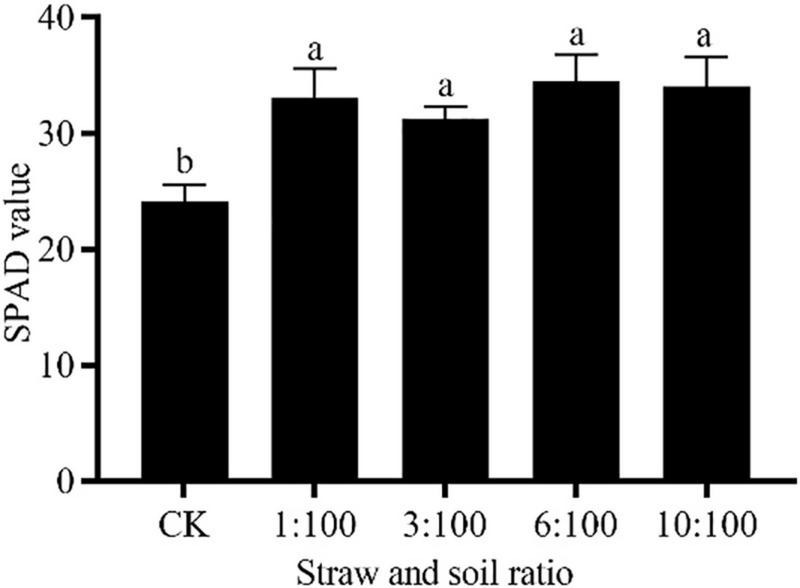
Effect of different ratios of milk vetch and soil on SPAD value of corn. All data were subjected to analysis of variance with *post hoc* LSD test. Different letters denote significant difference among different treatments at *P* < 0.05. Bar chart represents 5 replicates ± SD.

The participation of milk vetch can improve the PSII actual photochemical quantum efficiency (φPSII), electron transport rate (ETR), and photochemical quenching (qP). The change of φPSII and ETR reached a significant level among all the treatments, while the change of qP at 1:100 and 10:100 treatment reached a significant level. the maximal quantum yield of PSII (Fv/Fm) was improved at 3:100 and 6:100 treatment, and the change at 3:100 reached a significant level. All the treatments can decline the non-photochemical quenching (NPQ) of corn, and the changes reached a significant level. The lowest value of NPQ was recorded at 1:100 and 10:100 treatment (Supplementary Table 4). Milk vetch is a kind of green fertilizer with high nitrogen (N) nutrition, studies have found that the use of milk vetch can improve soil nutrition, especially N nutrition (Bimantara et al., 2020). The high-N condition was conducive to the photosynthesis parameters and maximal quantum yield of PSII (Yousuf et al., 2020). Considering that corn is a high-N plant, from the current experimental results, the high proportion of milk vetch is still beneficial to corn growth, and the follow-up experiments should be conducted around the straw returning threshold to prevent possible damage to corn (Figure 9). Incorporating the milk vetch as green manure showed a positive tendency on promoting the chlorophyll content of corn. In the study of corn straw on the growth of tomato seedlings, researchers found that the total chlorophyll and photosynthesis parameters were highest.

#### Different Ratios of Milk Vetch and Soil Treatment on Biochemical Properties of Corn

**FIGURE 10:**
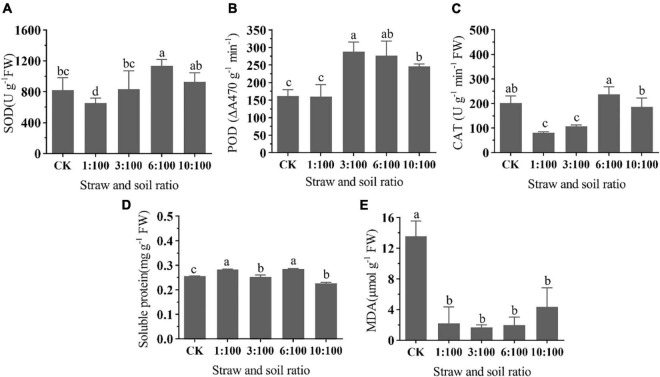
Effect of different ratios of milk vetch and soil on SOD activity **(A)**, POD activity **(B)**, CAT activity **(C)**, soluble protein **(D)**, and MDA **(E)** content of goosegrass. All data were subjected to analysis of variance with *post hoc* LSD test. Different letters denote significant difference among different treatments at *P* < 0.05.

The SOD and CAT activities (Figures 10A,C) decreased under the condition of low milk vetch proportion and increased with the increase of proportion. SOD activity reached a peak under 6:100 proportion. The SOD activity significantly improved at 6:100, while CAT activity significantly declined at low proportions (≤3:100). POD activity (Figure 10B) stands still at 1:100 treatment, and then significantly improved as the milk vetch proportion was greater than 3:100. In a similar study of “Effect of the aqueous extract of *Juglans. regia* leaves on the seed germination and seedling enzymatic activity of *Scutellaria baicalensis* in Shangluo,” the researcher found that a low concentration of aqueous extract promoted the protective enzyme activities of *Scutellaria baicalensis* seedling; but with the increasing concentration of aqueous extract, the promoting role gradually decreased (Peng, 2011). Previous studies on the effect of silicon application on sugarcane disease resistance found that silicon application can increase the activity of SOD and decrease the activity of POD in the sugarcane seedling stage, which resulted in the increase of MDA and H_2_O_2_, induced the resistance response, and finally enhanced the ability of disease resistance in the following growth stage (Deng et al., 2020). Garlic allelochemicals (aqueous extraction) act as plant biostimulants to enhance auxin biosynthesis and transportation, resulting in root growth promotion. Additionally, antioxidant enzymes activities regulations indicate activation of the defense responses in tomato seedlings resulting in better growth and development (Hayat et al., 2020), which is consistent with this study.

The 1:100 and 6:100 treatments significantly increased the content of soluble protein in corn (Figure 10D), while the content of soluble protein significantly declined at 10:100 treatment. As for the decrease of soluble protein content caused by the 10:100 treatment, it is speculated that the negative effect may be caused by high green manure content. Researchers found that four plant leaf extracts exhibited beneficial allelopathic effects on the onion plant protein content (Qari et al., 2020). All of the treatments can significantly decrease the content of corn MDA (Figure 10E). When the milk vetch proportion reached 10:100, MDA content tends to increase. Researchers of the previous studies about the allelopathic effect of co-culture pepper and garlic reported that a low garlic/pepper ratio tends to induce protective enzyme systems, reduce MDA content, and finally promote pepper growth, but a high garlic/pepper ratio resulted in a high concentration of garlic root exudates, which have deleterious effects on membrane lipid and inhibited protective enzyme activities (Ding et al., 2016). The tendency of protective enzyme systems activities, soluble protein, and MDA content in 10:100 treatment should be paid more attention. Further study should optimize the milk vetch returning proportion to provide a suitable condition for corn production.

This study provides positive evidence that milk vetch incorporated into the soil as green manure has the potential to inhibit the growth of weeds. Planting corn immediately after milk vetch is returned to the field, can suppress the emergence of weeds and promote the growth of corn. Combined with the weed control effect and the growth of corn, this study suggested that the ratio of milk vetch and soil should be within 10:100, and the ratio of 6:100 is the best under laboratory conditions.

## Data Availability Statement

The original contributions presented in the study are included in the article/Supplementary Material, further inquiries can be directed to the corresponding author/s.

## Author Contributions

SL and YM: conceptualization. SL: experimental method and design, software, original draft, and writing. YZ, ZC, and XD: data curation and revision of the manuscript. YM and ZM: funding acquisition, supervision, and validation. All authors have read and agreed to the published version of this manuscript.

## Conflict of Interest

The authors declare that the research was conducted in the absence of any commercial or financial relationships that could be construed as a potential conflict of interest.

## Publisher’s Note

All claims expressed in this article are solely those of the authors and do not necessarily represent those of their affiliated organizations, or those of the publisher, the editors and the reviewers. Any product that may be evaluated in this article, or claim that may be made by its manufacturer, is not guaranteed or endorsed by the publisher.
