# Supplementary material for: Astragalus sinicus Incorporated as Green Manure for Weed Control in Corn
Source: Front Plant Sci. 2022 Apr 29;13:829421. doi: 10.3389/fpls.2022.829421 (PMC9106406; doi:10.3389/fpls.2022.829421)
Supplement: Supplementary file 1 [file Table_1.DOCX]

**Table**

Table 1. Effect of different concentration milk vetch aqueous extract treatment on germination and growth of goosegrass (mean±SD; n=5)

| Treatment | Germination rate  (%) | Germination potential  (%) | Plant height  (mm) | Fresh weight  (mg) | Germination index | Vital index |
| --- | --- | --- | --- | --- | --- | --- |
| CK | 86.67±3.33 a | 83.33±4.08 a | 8.85±0.84 c | 42.91±6.35 ab | 42.98±1.42 a | 380.34±12.60 c |
| 2% | 80.67±6.41 b | 72.67±4.95 b | 11.73±1.64 b | 49.67±5.25 a | 41.55±2.63 a | 499.81±31.64 b |
| 5% | 80.00±4.08 b | 66.67±5.28 c | 12.88±1.41 a | 38.29±0.96 b | 39.26±1.08 b | 514.33±14.20 a |
| 30% | 28.67±3.80 c | 2.67±1.50 d | 11.34±1.50 b | 46.94±1.34 a | 7.70±1.23 c | 87.29±13.95 d |
| 80% | 0 d | 0 d | 0 d | 0 c | 0 d | 0 e |

† Numbers followed by the different letter within each column were significantly different at *P* < 0.05 by LSD test respectively.

Table 2. Effect of different milk vetch straw and soil ratios on the photosynthesis and fluorescence properties of goosegrass (mean±SD; n=15)

| Indicators | CK | 1:100 | 3:100 | 6:100 | 10:100 |
| --- | --- | --- | --- | --- | --- |
| Pn (μmol m-2 s-1) | 9.46±0.32 a | 3.29±0.53 c | 4.53±0.37 b | 3.82±0.40 c | 4.84±0.48 b |
| E (μmol m-2 s-1) | 0.84±0.21 a | 0.78±0.19 a | 0.46±0.15 c | 0.41±0.07 c | 0.62±0.17 ab |
| gtc (μmol m-2 s-1) | 40.33±10.24 a | 26.62±12.82 ab | 25.74±14.48 ab | 21.30±8.78 b | 20.88±9.86 b |
| Ci (μmol mol-1) | 107.61±16.84 d | 290.53±22.63 bc | 217.89±63.12 bcd | 174.11±37.02 d | 316.01±142.01 a |
| iWUE (μmol mmol-1) | 12.60±2.23 a | 4.13±0.93 b | 7.17±2.57 b | 7.03±2.32 b | 8.15±3.36 b |
| Fv/Fm | 0.71±0.019 a | 0.72±0.015 a | 0.73±0.012 a | 0.71±0.022 a | 0.72±0.021 a |
| φ PSII | 0.20±0.0027 b | 0.15±0.0048 e | 0.17±0.0069 d | 0.23±0.0088 a | 0.19±0.0058 c |
| ETR (μmol m-2 s-1) | 84.99±1.12 b | 62.9±2.03 e | 72.57±2.9 d | 98.76±3.69 a | 80.87±2.44 c |
| qP | 0.43±0.0077 b | 0.37±0.021 c | 0.41±0.041 bc | 0.54±0.056 a | 0.43±0.022 b |
| NPQ | 6.71±0.15 e | 16.6±0.10 c | 18.71±0.14 b | 26.26±0.27 a | 13.79±0.24 d |

† Net photosynthetic rate (Pn); transpiration rate (E); stomatal conductance (gtc); intercellular carbon dioxide concentration (Ci); instant water use efficiency (iWUE).

‡ Numbers followed by the different letter within each row were significantly different at *P* < 0.05 by LSD test respectively.

Table 3. Effect of different milk vetch straw and siol ratios on corn root system (mean±SD, n=5)

| Treatment | Total length(cm) | Average diameter(mm) | Surface area(cm^2^) | Volume(cm^3^) | Branch points | Root tips | Root activity(μg g^-1^) |
| --- | --- | --- | --- | --- | --- | --- | --- |
| CK | 654.47±165.71 a | 0.63±0.032 b | 131.80±37.55 b | 5.02±1.39 c | 4205.60±1003.09 a | 5081.60±441.25 c | 0.019±0.00011 e |
| 1:100 | 842.16±141.98 a | 0.73±0.038 a | 196.01±32.50 a | 8.60±1.04 a | 5069.00±1055.39 a | 6953.00±1213.71 ab | 0.021±0.00041 d |
| 3:100 | 685.75±186.76 a | 0.67±0.035 b | 136.18±35.59 b | 5.65±1.24 c | 3929.20±1195.40 a | 5391.60±873.95 bc | 0.035±0.00022 b |
| 6:100 | 722.32±106.03 a | 0.63±0.019 b | 146.47±16.57 b | 5.45±0.62 c | 4342.00±660.84 a | 6764.50±1060.61 abc | 0.079±0.0019 a |
| 10:100 | 652.51±37.95 a | 0.75±0.053 a | 156.24±7.43 b | 7.03±0.45 b | 4763.17±863.86 a | 7748.33±1840.36 a | 0.027±0.00047 c |

† Numbers followed by the different letter within each column were significantly different at P < 0.05 by LSD test respectively.

Table 4. Effect of different milk vetch and siol ratios on the photosynthesis and fluorescence properties of corn (mean±SD; n=15)

| Indicators | CK | 1:100 | 3:100 | 6:100 | 10:100 |
| --- | --- | --- | --- | --- | --- |
| Pn (μmol m-2 s-1) | 12.70±2.72 b | 12.93±2.90 b | 16.81±2.25 a | 16.53±0.89 a | 16.08±2.89 a |
| E (μmol m-2 s-1) | 1.11±0.15 b | 1.05±0.20 b | 1.43±0.14 a | 1.42±0.13 a | 1.42±0.26 a |
| gtc (μmol m-2 s-1) | 49.96±7.60 b | 51.11±10.25 b | 67.83±7.78 a | 68.98±7.60 a | 67.81±12.78 a |
| Ci (μmol mol-1) | 125.88±26.89 a | 127.08±13.66 a | 126.14±13.93 a | 132.59±21.61 a | 137.06±8.05 a |
| iWUE (μmol mmol-1) | 11.37±1.09 b | 12.20±0.74 a | 11.70±0.74 b | 11.71±0.87 b | 11.33±0.33 b |
| Fv/Fm | 0.65±0.027 ab | 0.64±0.034 b | 0.67±0.025 a | 0.66±0.038 ab | 0.65±0.020 ab |
| φ PSII | 0.21±0.026 d | 0.31±0.0069 ab | 0.30±0.0071 b | 0.27±0.022 c | 0.32±0.019 a |
| ETR (μmol m-2 s-1) | 97.13±11.93 d | 143.61±3.20 ab | 140.09±3.30 b | 126.72±10.16 c | 148.95±8.97 a |
| qP | 0.52±0.045 b | 0.64±0.014 a | 0.63±0.011 b | 0.63±0.018 b | 0.66±0.024 a |
| NPQ | 3.02±0.35 a | 1.79±0.24 d | 2.23±0.13 c | 2.46±0.16 b | 1.82±0.34 d |

† Net photosynthetic rate (Pn); transpiration rate (E); stomatal conductance (gtc); intercellular carbon dioxide concentration (Ci); instant water use efficiency (iWUE).

‡ Numbers followed by the different letter within each row were significantly different at *P* < 0.05 by LSD test respectively.
